# Imaging Evidence for Cerebral Hyperperfusion Syndrome after Intravenous Tissue Plasminogen Activator for Acute Ischemic Stroke

**DOI:** 10.1155/2016/8725494

**Published:** 2016-05-03

**Authors:** Yi Zhang, Abhay Kumar, John B. Tezel, Yihua Zhou

**Affiliations:** ^1^Department of Neurology, Saint Louis University, Saint Louis, MO 63110, USA; ^2^Department of Radiology, Saint Louis University, Saint Louis, MO 63110, USA

## Abstract

*Background*. Cerebral hyperperfusion syndrome (CHS), a rare complication after cerebral revascularization, is a well-described phenomenon after carotid endarterectomy or carotid artery stenting. However, the imaging evidence of CHS after intravenous tissue plasminogen activator (iv tPA) for acute ischemic stroke (AIS) has not been reported.* Case Report*. Four patients were determined to have manifestations of CHS with clinical deterioration after treatment with iv tPA, including one patient who developed seizure, one patient who had a deviation of the eyes toward lesion with worsened mental status, and two patients who developed worsened hemiparesis. In all four patients, postthrombolysis head CT examinations were negative for hemorrhage; CT angiogram showed patent cervical and intracranial arterial vasculature; CT perfusion imaging revealed hyperperfusion with increased relative cerebral blood flow and relative cerebral blood volume and decreased mean transit time along with decreased time to peak in the clinically related artery territory. Vascular dilation was also noted in three of these four cases.* Conclusions*. CHS should be considered in patients with clinical deterioration after iv tPA and imaging negative for hemorrhage. Cerebral angiogram and perfusion studies can be useful in diagnosing CHS thereby helping with further management.

## 1. Introduction

Cerebral hyperperfusion syndrome (CHS) is a rare but expected complication after carotid endarterectomy or carotid artery stenting. Incidence of CHS is 1–3% after carotid endarterectomy [1]. CHS after iv tPA in patients with acute ischemic stroke (AIS) has also been recognized [2, 3]. However, radiographic evidence of CHS has not been described. We present this case series with imaging evidence of cerebral hyperperfusion after iv tPA treatment for AIS.

## 2. Case Report

We reviewed a total of 772 CT perfusion (CTP) studies performed in our hospital from July 2009 to October 2015 to identify AIS patients who developed CHS after intravenous thrombolysis treatment. CTP hyperperfusion was characterized by increased relative cerebral blood flow (rCBF) and relative cerebral blood volume (rCBV) and decreased mean transit time (MTT) along with decreased time to peak (TTP) in the clinically related artery territory. Cerebral hyperperfusion syndrome was characterized by focal neurological deficit or seizures developing after cerebral revascularization. Revascularization was confirmed by CT angiogram (CTA). The study was approved by our Institutional Review Board.

We identified four cases of CHS after iv tPA for AIS (Table 1).

**Case 1 .**

**Figure 1:**
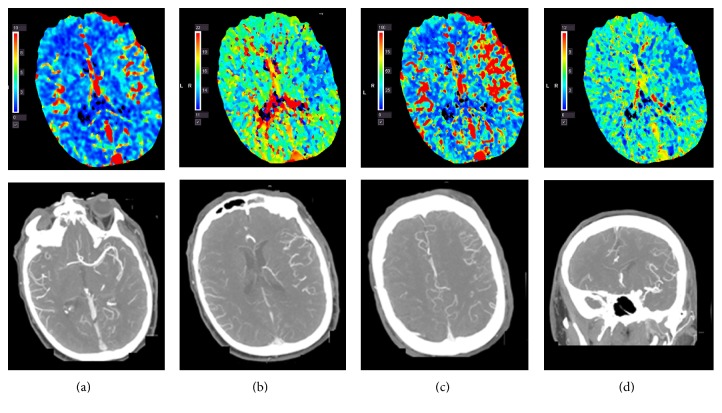
Hyperperfusion on computed tomography perfusion and the dilation of the left middle cerebral artery and its branches on CT angiogram after iv tPA treatment: (a) increased cerebral blood volume; (b) shortened time to peak; (c) increased cerebral blood flow; (d) shortened mean transit time.

**Figure 2:**
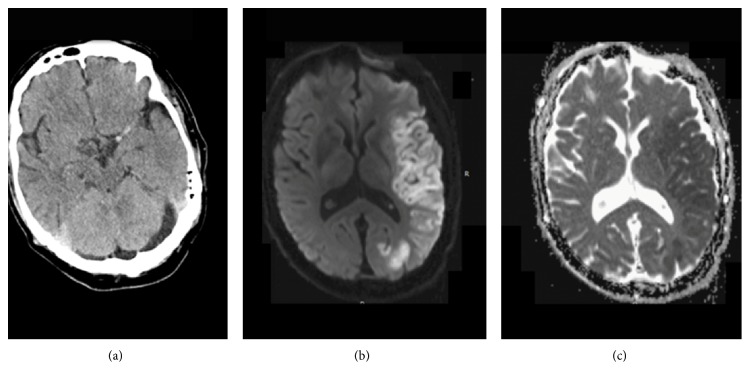
Hyperdense middle cerebral artery on the initial CT prior to intravenous tissue plasminogen activator (a). Diffusion-weighted magnetic resonance imaging (b). Apparent diffusion coefficient (c) confirms acute infarction in the left MCA territory.

A 61-year-old Caucasian male with history of chronic lymphocytic leukemia in remission and recurrent sinusitis was found in his garage with right-sided weakness. Examination at the local hospital 2 hours after the onset of the symptoms was significant for aphasia and right hemiplegia. Computed tomography of the head suggested a hyperdense left middle cerebral artery (MCA). He was given iv tPA three hours after onset of the symptoms and then transferred to our hospital for further management. His blood pressure was 157/82 mmHg. En route, the patient developed one seizure-like episode with sudden onset jerking movements of all extremities. Upon arrival, he was mute, confused, and agitated; however he was able to move the four extremities symmetrically. His National Institutes of Health Stroke Scale (NIHSS) was 9. The patient underwent CTA and CTP in anticipation of mechanical thrombectomy upon arrival. Instead, CTA showed patent cervical and intracranial arterial vasculature and a dilated left MCA while CTP revealed an increased rCBF and rCBV in the left middle cerebral artery (MCA) territory as well as a decreased MTT and TTP (Figure 1). MRI of the brain performed the following day demonstrated a large acute MCA territory infarct (Figure 2). Further workup revealed evidence of vegetation on the anterior and posterior mitral valve leaflets, likely the source of emboli. The patient was empirically treated with antibiotics for infective endocarditis. He was discharged to a rehabilitation facility on hospital day 6 with NIHSS 2. At the 3-month follow-up visit, the patient's right-sided weakness had resolved although aphasia persisted.

**Case 2 .**

An 82-year-old Caucasian male with history of prostate cancer status after prostatectomy, hypertension, and atrial fibrillation developed sudden onset right upper extremity weakness and dysarthria. His warfarin was discontinued a week before for a right knee partial arthroplasty done two days prior to stroke onset. NIHSS was 9 at the local emergency department for which he received iv tPA and was then transferred to our hospital. The patient's blood pressure was 133/62 mmHg. His dysarthria resolved after iv tPA although his right upper extremity strength got worse on hospital day 2. A stat CTA showed no evidence of vessel occlusion but demonstrated dilated left MCA branches. CTP revealed hyperperfusion of the left MCA territory while noncontrast CT showed a left basal ganglia infarct. The patient also had urinary tract infection at admission, which was treated with antibiotics. The patient remained stable thereafter and improved with an NIHSS of 7 at the time of discharge.

**Case 3 .**

A 92-year-old male with history of atrial fibrillation and colon cancer developed right-sided weakness, global aphasia, and confusion. His NIHSS was 23. The patient received iv tPA. The patient's right-sided weakness worsened immediately following the infusion. His blood pressure was 143/76 mmHg. He underwent CTA and CTP, which demonstrated increased cerebral blood perfusion involving the left MCA territory. Noncontrast CT head 24 hours after thrombolysis showed infarction of a cortical area of the left parietal lobe. The patient had bright red blood per rectum one time. His vital signs and hemoglobin had been normal until hospital day 2 when he developed acute myocardial infarction. The family decided on palliative care, considering the patient's preexisting illness and age. The patient was deceased on hospital day 9.

**Case 4 .**

A 65-year-old female with history of hypertension, deep venous thrombosis, and colitis developed right-sided weakness and expressive aphasia. She was brought to the local hospital. She was awake and could not respond appropriately. Her NIHSS was 20. Stat CT head revealed a hyperdense left MCA. The patient received iv tPA and subsequently was transferred to our hospital. Her blood pressure was 136/79 mmHg. En route, the patient developed a deviation of the eyes toward the left. She became less arousable. The patient became mute upon arrival. A stat CTA showed patent cervical and intracranial arterial vasculature and a dilated left MCA branch. CTP revealed large area of hyperperfusion on the left hemisphere. Follow-up MRI of the brain performed the following day demonstrated a large acute left MCA territory infarct, which matched the large hyperperfusion area seen on the CTP. During the hospitalization, strength on the right side improved significantly, although global aphasia remained. The patient was discharged to an acute rehabilitation facility at day 5 with NIHSS 10.

## 3. Discussion

In this case series, we present 4 cases of CHS with radiographic evidence after iv tPA treatment for AIS. CTA and CTP examinations after iv tPA treatment demonstrated recanalization and dilation (in three of the four cases) of the affected intracranial arteries as well as increased perfusion to the regions of the infarcted brain. Therefore, in patients with clinical deterioration after iv tPA treatment of acute ischemic stroke, cerebral CTA and CTP examinations may be helpful to diagnose cerebral hyperperfusion syndrome and provide evidence to guide further management.

Cerebral hyperperfusion syndrome is a rare complication following rapid revascularization. The likely pathophysiology is impaired cerebral autoregulation leading to increased cerebral blood flow, above the metabolic demands of brain tissue [4]. However, knowledge of CHS remains limited. Preexisting disease, such as chronic hypoperfusion from artery stenosis, infection, and chronic inflammatory disease, as well as expression of genes might affect cerebral myogenic, metabolic, or neurogenic regulation. Deficits are usually cortical which may be new or may represent worsening of a preexisting neurological deficit. In the study, three of the four cases had cortical infarction. Seizures may present as focal or generalized, depending on the affected cortical area [5]. One case had generalized seizure.

Cerebral hyperperfusion syndrome is a clinical syndrome of reperfusion injury which can occur in numerous ways including activation of endothelium, excess production of oxygen free radicals, inflammatory responses and leukocyte recruitment, increase in cytokine production, and edema formation [6]. There is disruption of the blood-brain barrier (BBB) through the release of neutrophil-derived oxidants and proteolytic enzymes. Two of the four cases in this study had active infectious disease during thrombolysis. One case had chronic colitis. Hyperperfusion initializes an inflammatory cascade, resulting in the deterioration of salvageable penumbra [7]. Infection appears to be an important trigger that precedes ischemic strokes and can bring about irreversible injury through a range of potential mechanisms. Preexisting infection in our patients may be a contributing factor for the development of CHS.

Interestingly, three of the four cases in the study had history of cancer, which may have contributed to impaired cerebral autoregulation. Tumor endothelial cells often lose their normal barrier function. Changes in endothelial shape result in intercellular gaps or holes that leak fluid, blood, and fibrin into the surrounding tissue [8]. The vascular endothelium is a dynamic cellular “organ” that controls passage of nutrients into tissues, maintains the flow of blood, and regulates the trafficking of leukocytes. Tumor blood vessels have irregular diameters, are fragile and leaky, and have abnormal blood flow [9]. The pathophysiology of cerebral autoregulation in patients with cancer history needs further investigation.

Our case series is limited by the lack of angiographic and perfusion studies prior to thrombolysis, although the presence of hyperdense MCA sign (as in Cases  1 and 4), sudden onset of focal neurological deficits, and high NIHSS suggest large vessel involvement. We were also not able to assess cerebral autoregulation based on the autoregulation index to monitor cerebral blood flow regulation. This would require a more specialized setting to do so. All four cases presented with a left hemisphere stroke. This might be due to reporting bias. A patient with a right hemisphere stroke commonly has neglect, which might mask the clinical deterioration.

A main concern for clinical deterioration following tPA administration has been hemorrhagic transformation which can be evaluated with a noncontrasted head CT. One study showed that early neurological deterioration without clear mechanism affected 7% of the patients with acute stroke [10]. We believe some of these cases may be due to CHS, which is currently underrecognized and may also be delayed in onset as what happened in Case 2. The true incidence of CHS may thus be underreported as well given lack of angiographic and perfusion studies in the post-tPA administration setting. Therefore, when clinical deterioration following iv tPA cannot be simply explained by other obvious reasons such as hemorrhage, CHS should be considered. In addition, early clinical deterioration (or deterioration after improvement) can occur in ischemic stroke patients who have not received iv tPA treatment, which could be attributed to a number of reasons, including hemorrhagic transformation, internal herniation due to mass effect, and ventricular entrapment. However, as early spontaneous thrombolysis can occur, early clinical deterioration can potentially be due to CHS, when other causes are not apparent.

## 4. Conclusion

The report provides imaging evidence of hyperperfusion in patients with CHS after iv tPA for acute cerebral infarction. Therefore, cerebral CTA and CTP studies should be considered to confirm the presence of cerebral hyperperfusion in patients with clinical deterioration after tPA treatment for acute stroke.

## Figures and Tables

**Table 1:** Four cases of cerebral hyperperfusion syndrome after intravenous tPA for acute ischemic stroke.

	Age	Sex	Stroke symptoms	PMH	NIHSS (onset)	tPA dose	CHS symptoms	NIHSS (D/C)	Infarct area
Case 1	61	M	Aphasia, right hemiplegia	CLL	9	90 mg	Seizure	2	Cortical
Case 2	82	M	Dysarthria, right hemiplegia	Prostate cancer. HTN, Afib	9	70 mg	Worsening hemiplegia	7	Basal ganglia
Case 3	92	M	Aphasia, right hemiplegia, confusion	Colon cancer, Afib	23	74 mg	Worsening hemiplegia	Deceased	Cortical
Case 4	65	F	Aphasia, right hemiplegia	HTN, DVT, colitis	20	90 mg	Eyes deviation, confusion	10	Cortical

PMH: past medical history; NIHSS: NIH stroke scale; CHS: cerebral hyperperfusion syndrome; D/C: at discharge time; M: male; F: female; CLL: chronic lymphocytic leukemia; HTN: hypertension; Afib: atrial fibrillation; DVT: deep vein thrombosis.
